# A Systematic Review of Beef Meat Quantitative Microbial Risk Assessment Models

**DOI:** 10.3390/ijerph17030688

**Published:** 2020-01-21

**Authors:** Vincent Tesson, Michel Federighi, Enda Cummins, Juliana de Oliveira Mota, Sandrine Guillou, Géraldine Boué

**Affiliations:** 1 INRA, Oniris, SECALIM, 44307 Nantes, France; vincent.tesson2@inra.fr (V.T.);; 2 Biosystems Engineering, School of Agriculture, Food Science and Veterinary Medicine, Agriculture and Food Science Centre, University College Dublin, Belfield, Dublin 4, Ireland

**Keywords:** food safety, meat, beef processing, farm-to-fork, mathematical model, predictive microbiology, QMRA

## Abstract

Each year in Europe, meat is associated with 2.3 million foodborne illnesses, with a high contribution from beef meat. Many of these illnesses are attributed to pathogenic bacterial contamination and inadequate operations leading to growth and/or insufficient inactivation occurring along the whole farm-to-fork chain. To ensure consumer health, decision-making processes in food safety rely on Quantitative Microbiological Risk Assessment (QMRA) with many applications in recent decades. The present study aims to conduct a critical analysis of beef QMRAs and to identify future challenges. A systematic approach, the Preferred Reporting Items for Systematic Reviews and Meta-Analyses (PRISMA) guidelines, was used to collate beef QMRA models, identify steps of the farm-to-fork chain considered, and analyze inputs and outputs included as well as modelling methods. A total of 2343 articles were collected and 67 were selected. These studies focused mainly on western countries and considered *Escherichia coli* (EHEC) and *Salmonella* spp. pathogens. Future challenges were identified and included the need of whole-chain assessments, centralization of data collection processes, and improvement of model interoperability through harmonization. The present analysis can serve as a source of data and information to inform QMRA framework for beef meat and will help the scientific community and food safety authorities to identify specific monitoring and research needs.

## 1. Introduction

Microbial foodborne disease is a major concern in terms of public health due to the high risk of microbial contamination of foods by several types of biological hazards. The World Health Organization (WHO) estimated that in 2010, at a global scale and annually, more than 580 million illnesses and 351,000 deaths were associated with food contaminated by enteric pathogens [1]. Amongst the foods, meat and meat-based products are considered as one of the main vehicles of these pathogens that accounted for more than 16% of total foodborne outbreaks in Europe in 2016 [2]. More precisely in France, a study in progress [3] estimated that the burden of disease associated with beef consumption (due to six bacteria, one virus, and one parasite) was 177,610 [95% CI = 93,890–313,680] illnesses per year among 67 million people, including very mild and severe effects. This corresponds to 2840 [95% CI = 1680–4240] Disability Adjusted Life Years (DALY). The most frequent pathogen identified was *Campylobacter* spp., responsible for 66,140 [95% CI = 17,410–152,000] foodborne illnesses cases and 1000 [95% CI = 310–1860] DALY. The most severe pathogens were *Brucella* spp. and *Toxoplasma gondii* parasite with 0.4 [95% CI = 0.0–1.7] and 0.3 [95% CI = 0.1–0.6] DALY per case, respectively.

A risk-based food safety management approach was recommended and adopted in the last 20 years through intergovernmental organizations like the Codex Alimentarius Commission (CAC) [4,5,6,7] including risk assessment, risk management, and their associated communication. Microbial risk assessment is a scientific evaluation that aims to provide an estimation of a risk considering the probability and the severity of health effects caused by a hazard in order to support decision-making processes. It consists of four steps: hazard identification, exposure assessment, hazard characterization, and risk characterization. Quantitative Microbial Risk Assessment (QMRA) was first used for water and was then extended to diverse foodstuffs [8,9,10,11]. QMRA starts with the design of the risk question in order to determine the situation by identifying the hazard, the contaminated matrix, the population concerned, and its exposure [12]. Then the model used for the assessment needs to be designed and populated with both data and knowledge regarding effects of the processes undergone by the food matrix as well as the response of the pathogen to them, in terms of either growth or inactivation. Using QMRA models enables experts to estimate the risk to which the population may be exposed, evaluate possible risk mitigation strategies, and generate knowledge for better management of risks associated with contamination events.

Although QMRA is now well established and developed, a large heterogeneity exists between all QMRAs performed even when considering the same matrix-pathogen combination. It is well recognized that resources and knowledge generated by one QMRA are difficult to reuse and apply with another one [13].

In the case of risk assessments applied to beef meat, estimating the impact of a contamination by a pathogen may require knowledge of the whole farm-to-fork chain since contamination may occur at different stages. Considering existing data and knowledge gaps, it is therefore essential to be able to transpose results from one situation to another, for example concerning similar steps of growth or inactivation of a pathogen on a similar matrix of interest. This transposition is not always straightforward, and there is a clear need to harmonize QMRA models [13].

The objective of the present paper is to perform a critical analysis of existing QMRAs of beef meat along the farm-to-fork chain. A systematic approach was used to collect available beef QMRAs performed to date. These studies were synthesized and methods used were analyzed. This work will provide to scientists and risk managers an overview of current beef QMRAs studies available with an insight on gaps identified.

## 2. Materials and Methods

A literature search was conducted to collect exhaustively and analyze risk assessments related to beef-based products. The search was performed following PRISMA guidelines [14,15] on Web of Science and Scopus databases. For each database, the following search queries were used:

Web of Science: TITLE: (beef OR cattle OR boeuf) AND TITLE: (risk* OR risque* OR “risk assessment” OR aqr OR qmra OR exposure OR “model$ing”)
Scopus: TITLE (beef OR cattle OR bœuf) AND TITLE (risk* OR risque* OR “risk assessment” OR aqr OR qmra OR exposure OR modeling OR modelling)

**Figure 1:**
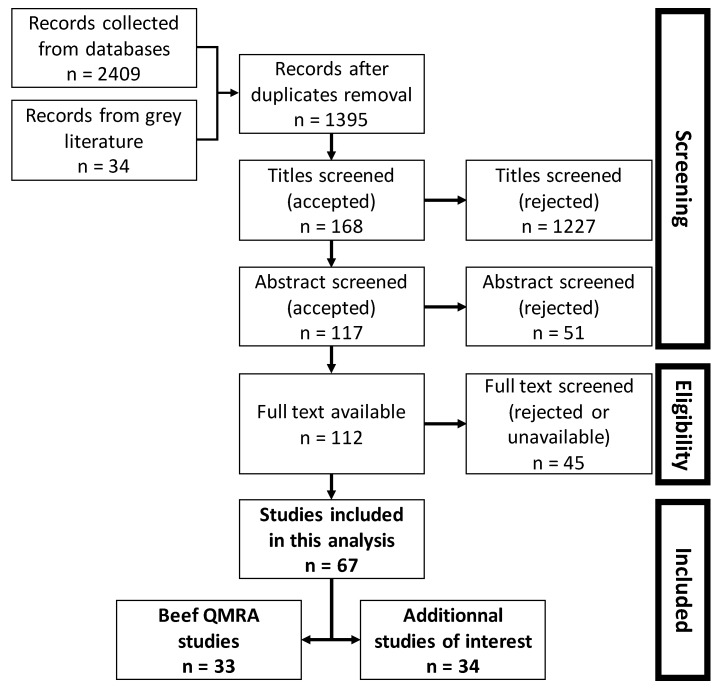
Flow chart of studies selected using Preferred Reporting Items for Systematic Reviews and Meta-Analyses (PRISMA) method. QMRA: Quantitative Microbial Risk Assessment.

The search was done on each database considering their respective starting year—1956 for Web of Science and 1788 for Scopus—to the date of the search, which was 15 February 2019. A total of 2409 articles (1210 from Web of Science and 1199 from Scopus) were collected, and 34 papers from the grey literature (mostly reports from European health agencies and WHO) and additional papers recommended by experts (Figure 1). Duplicates were removed and then articles were screened for titles, abstracts, and full texts, rejecting articles that did not comply with the following criteria: dealing specifically with beef QMRA and being an original research article. Papers selected for the present review dealt with beef products and the following specific topics: microbial hazards, risk assessment, predictive microbiology, estimation of microorganism prevalence, microbial tracking, or heat and mass transfer modelling.

## 3. Results

Papers screened using the PRISMA method enabled the collection of a total of 67 papers (Figure 1). Within this selection, 33 papers concerned beef meat QMRA [16,17,18,19,20,21,22,23,24,25,26,27,28,29,30,31,32,33,34,35,36,37,38,39,40,41,42,43,44,45,46,47,48] and 34 were on specific aspects that can be of interest in beef QMRA models development [49], predictive microbiology models [50,51,52,53,54,55,56,57,58,59,60,61,62,63,64,65,66,67,68,69,70,71,72,73], the estimation of the prevalence of pathogens at different steps [74,75], microbial tracking [76], and heat and mass transfer models [77,78,79,80,81,82].

### 3.1. Risk Question

Studies essentially covered both beef meat and meat preparations to be eaten raw (e.g., tartare), cooked (ground meat and burgers), but also carcasses as well as specific vectors of contamination like water and even the environment itself [26] (Table 1). Collected publications concerned mainly European populations (15 papers), with a focus on the French (*n* = 5) and UK (*n* = 6) ones, along with North American populations (*n* = 9) and other countries (Australia, Brazil, Chile, Argentina, Zambia, and South Korea). On several occasions, assessments that integrated the meat chain up to the consumption step mainly focused on the population under 15 years (*n* = 3) while the majority designed their study to cover the whole consumer population (*n* = 23).

The objective of these studies was to assess:

− risks of illness and health burden associated with consumption of beef meat and preparations [16,21,22,24,25,32,33,34,35,36,38,39,42,44];
− the effect of mitigation interventions [16,27,28,36,39,40,43,47,48];
− all or part of the farm-to-fork chain [18,20,24,29,30,31,37];
− transmission pathways or contribution to the public health burden [17,26,43,46];
− impact of manufacturing and consumption practices [23];
− investigation on outbreaks [41].

### 3.2. Identification of Main Hazards in Beef Products

**Figure 2:**
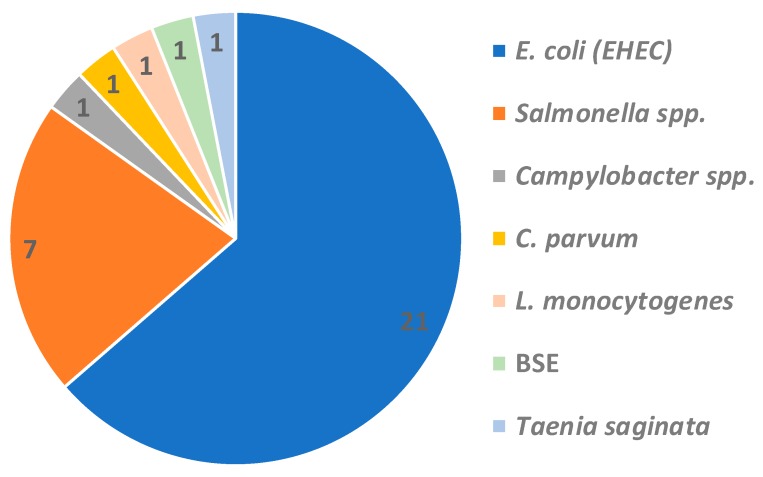
Pathogens considered in beef QMRA. EHEC: Enterohemorrhagic *E. coli*; BSE: Bovine Spongiform Encephalopathy.

The first step of QMRA consists of the identification and selection of hazards to be assessed. As revealed through collected papers, a majority of studies (21/33) dealt with *Escherichia coli* — mostly Enterohemorrhagic *E. coli* (EHEC), but also wild types with antibiotic resistance [19] — contaminations followed by *Salmonella* spp. (7/33). Other studies concerned *Listeria monocytogenes, Campylobacter* spp., *Cryptosporidium* spp., Bovine Spongiform Encephalopathy (BSE), and *Taenia saginata* (1/33 each) (Figure 2).

Issues related to EHEC, *Salmonella* spp., and *Campylobacter* spp. may be tightly associated with contamination events at the farm and from intestinal contents (fecal and hide cross-contamination) [83,84]. With regards to infecting patients, outcomes ranged from gastroenteritis, for EHEC, *Campylobacter* spp. and *Salmonella* spp., to human uremic syndrome for several EHEC strains. EHEC contamination of food may also result in the production of bacterial toxins which may lead to severe or even lethal outcomes. *L. monocytogenes* are associated with bacterial growth problems occurring during storage, even at low temperatures. The population at risk consists of infants, pregnant women, elderly, and immunocompromised people highly susceptible to listeriosis. Other hazards like *Clostridium perfringens*, *Yersinia* spp. for example still exist, but no documented QMRA studies were retrieved through the exhaustive analysis protocol.

### 3.3. Exposure Assessment

**Figure 3:**
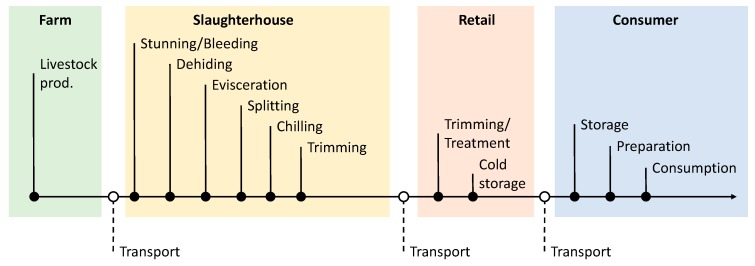
Farm-to-fork chain for beef meat as commonly considered by QMRA studies.

Following QMRA steps, exposure assessment corresponds to the quantification of the amount of microorganisms ingested by humans through food by combining hazard levels in food with consumer food intake quantity [13]. Hazard levels in food can be estimated through sampling campaigns and microbiological analysis or by predictive microbiology from raw materials up to the end-product consumption [85]. Thus, according to the QMRA scope, the model may cover all or part of the “farm-to-fork” meat chain of the beef product assessed (Figure 3).

**Figure 4:**
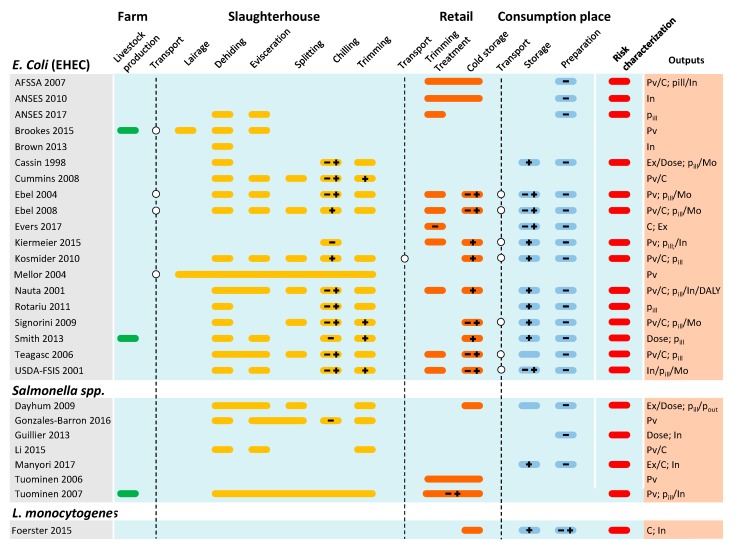
Meat chain steps considered in collected beef QMRAs for Enterohemorrhagic *E. coli* (EHEC), *Salmonella* spp., and *Listeria monocytogenes (*−: bacterial reduction accounted, +: bacterial growth accounted; Ex: exposure; Pv: prevalence; C: count and/or concentration; p_ill_: probability of illness; p_out_: probability of outbreak; Mo: mortality; DALY: disability-adjusted life year expectancy).

A graphical synthesis of steps specifically considered in each QMRA collected on EHEC, *Salmonella* spp., and *Listeria monocytogenes* has been done in Figure 4. Considering the collated papers, several steps have been overshadowed in Figure 4 as they were out of the scope of the study, seemed to have no impact on the contamination levels of the products, or were too difficult to model due to a high number of factors to consider. Steps that were almost systematically skipped when modelling are transportation and stunning steps at the slaughterhouse. Three transportation steps occur, one of animals from farm to slaughterhouse and the others of the products from industry to retail and to consumer house. When considered by authors, transportation of live animals may cause a possible increase in the prevalence of infected animals in the truck [27,34]. Brookes et al. [27] modelled live animal transport, and the QMRA carried out by the US Department of Agriculture-Food Safety and Inspection Service (USDA-FSIS) [34] considered after a review of the available literature that the transport of potentially infected live animals did not present a risk of an increase in fecal prevalence, despite its duration. The same assumption was also made by Signorini and Tarabla [35]. The same omission goes for the hide prevalence of animals, mainly due to lack of data and the fact that fecal prevalence appeared as a better indicator of contamination [34,35]. Concerning transport of meat products to retail outlet and home, this step was considered as having no significant impact on the contamination, because most of the time the storage temperature was well controlled [34] or its elimination was for simplification purposes [35]. To do this, authors assumed that the transit time between the slaughterhouse and the retail outlet, and then the home, was generally too short to allow microbial growth, particularly when considering the lag time before growth after temperature changes. Lairage at the slaughterhouse was considered as being not relevant in collected studies when assessing its impact on contamination of animals. Stunning of animals is also considered as a step to omit as it will not contribute to a significant contamination to the animal. Therefore, the report from the USDA-FSIS emphasizes that, despite the lack of data concerning the hide prevalence, the fall of the animal body on the floor after the passage of a contaminated animal may bring a risk of hide contamination [34].

#### 3.3.1. Farm: Livestock Rearing

Livestock rearing corresponds to the most upstream part of the meat chain with the on-farm production and the transport to slaughterhouse. Importation of animals from foreign countries may also have to be considered to model the production step [25,39,40]. As mentioned above, transport from farm to slaughterhouse is often excluded from assessments, but some authors tried to model contaminations during transport [18,24,27,33]. On a broader point of view, most of the studies focused only on EHEC contamination, leading to data gaps concerning other pathogens [23].

The farm part corresponds to the starting point of a high number of bottom-up models as the very first contamination of animals may occur at this point [16,18,24,27,29,34,35,36,43]. This contamination may be the main one and drive the subsequent contamination of other animals on the farm during transport or lairage, as well as potentially leading to the contamination of carcasses and meat through self- and cross-contamination [18,24,36]. For example, Smith et al. [36] identified the most effective risk mitigation strategy to be a vaccination against EHEC prior to the processing step in order to reduce the pathogen prevalence in Canadian ground beef and cuts.

Data collection on prevalence and/or concentration of pathogens in beef during the farm stage was highlighted as a difficult task [32]. Breeding practices, location, seasonal prevalence of asymptomatic shedders, and heterogeneity of prevalence and concentration within the herd affect the transmission dynamics, leading to difficulties in estimating the real prevalence of contaminated animals [27,32,86]. Some authors conducted sampling campaigns to approximate prevalence of the contaminations of interest at an early stage of carcass processing [16,23,24,26,29,30,31,32,33,40,42,87]. For the purposes of their exposure assessment, Teagasc [32] commissioned a study to estimate the prevalence of EHEC contamination in Irish living cattle, with a result of 109 contaminated hide samples on 1500 (7.3%) [88]. Dayhum [42], during a sampling campaign at a French slaughterhouse, estimated the prevalence of *Salmonella* spp. in beef feces to be 9.12% (27 contaminated sample out of 296). Based on these original data and often for a specific country, other studies tried to model a mean country prevalence [34,48].

A small number of collected papers described the farm part, a critical step where interventions could be conducted to reduce the final contamination of meat and meat products [16,20,24,26,27,30,31,32,34,35,36,43]. For instance, Rotariu et al. [26] stated that working on reducing the high shedders proportion within the lot or simply reducing the shed concentration may be a requirement to achieve a halving of human EHEC illnesses. The possibility of conducting large-scale vaccination campaigns, as illustrated by Brookes et al. [27], has been investigated but still has a limited effectiveness. Moreover, contamination will continue to occur during the steps of transportation and lairage following variability in vaccination and probiotics efficacy, as well as contact with cattle from multiple farms [27], but are commonly not considered.

#### 3.3.2. Slaughterhouse

Slaughtering is one of the most documented steps in the meat chain according to our research. This results from the monitoring of numerous parameters of the processing steps done by meat manufacturers and the fact that most of these steps are rather common between processing plants from the same country due to national and international guidelines regarding meat handling and processing [89,90].

Meat processing appears as the second most significant contributor to the final contamination of meat and meat products. This is mainly due to cross-contamination from hide and gastro-intestinal tract content leakages during dehiding and evisceration, which can lead to secondary contaminations between carcasses or through contact with equipment during carcass splitting and trimming [86]. Note that secondary contamination is an event that may be neglected by some authors as they may state that it is of non-significant impact on final prevalence or concentration or was difficult to model [17]. Processing steps known to introduce contaminations into the meat processing chain are the dehiding and the evisceration steps.

Contamination during dehiding is linked with hide-to-carcass transfers during dirty surface spots removal [20,26,27,30,31,32,37,43], through dirt-spreading during hide removal, or the rupture of surface lymph nodes [43]. Gonzales-Barrón et al. [37] estimated through meta-analysis the mean prevalence of carcasses contaminated by EHEC after dehiding to be 4.8% (SD: 0.014) when the same data after bleeding was 12.5% (SD: 0.011).

Evisceration is one of the most critical processing steps; if it is not properly done it can lead to the rupture of the gastrointestinal tract (GIT) and the release of its content. Due to its extremely high bacterial load, equal to that of feces, a spotted GIT content leakage may result in the carcass being systematically consigned [28]. Most models take the evisceration step into account [17,24,27,30,31,33,36,37,43]. For example, Ebel et al. [33] assumed that contamination of carcass through gut rupture might happen for zero to two percent of carcasses. Cummins et al. [31] considered for their part that the contamination resulting from evisceration should be at the same level as that resulting from dehiding.

As already observed by Brookes et al. [27] considering dehiding, contamination of carcasses by GIT greatly influences the prevalence of pre-chilled carcasses according to fecal bacterial prevalence and loads. On the other hand, Kosmider [17], based on works from Cummins et al. [31], assumed that carcass prevalence following dehiding or evisceration remains quite the same, while Gonzales-Barrón et al. [37] concluded that the possible bacterial concentration increase during evisceration may not be fully balanced by the decrease achieved through the succession of the dehiding, rinsing, and chilling steps.

At the end of the processing chain, freezing/chilling are crucial steps [16,17,20,24,26,30,31,32,33,34,35,36,42,48] with the purpose to avoid pathogen growth within and on the carcasses in order to maintain meat safety. Indeed, from the initial stunning of animals to their evisceration, the muscle temperature remains around 35 °C. Most HACCP (Hazard Analysis Critical Control Point) plans consider this step as a microbial development control measure and tend to shift surface temperature below 4°C within 24 h after animal death [91].

For this step, many studies integrate predictive microbiology models that may account for a possible decrease occurring during chilling [16,17,20,24,25,26,30,32,33,34,35,36,37] but more importantly for the growth that may occur during chilling and storage [16,17,20,24,26,30,31,32,33,34,35]. No contamination risk during this step emerged from the collected papers, as some authors estimate that the main contamination sources are in the upstream [18]. On the other side, several authors consider that chilling of carcasses have no significant effect on their bacterial count [35] and may even see the contamination increase [32]. This statement may vary with the chilling method as Smith et al. [36] showed that the use of water spray chilling may increase the risk of illness associated with ground beef while this method can reduce the EHEC count on carcasses, as aerosols may spread to other carcasses and increase the pathogen prevalence. In the same way, Gonzales-Barrón et al. [37] observed that while pre-chill washing may reduce *Salmonella* spp. prevalence in Brazilian beef from 8.6% to 7.5%, a pathogen spreading risk still remains.

#### 3.3.3. Post-Slaughtering: Processing and Retail

Accounting for post-processing handling highly depends on the scope and products covered by the QMRA, for example ground meat [16,17,21,22,23,24,25,29,30,33,34,35,36,42]. Here, post-processing corresponds to handling occurring from the processing of the carcass followed by distribution to the consumer. This also accounts for the manufacturing of products like burgers, for example.

Carcass transformation corresponds to the trimming of all relevant meat parts along with their processing—for example, mincing. This step is acknowledged as being a significant source of contamination and cross-contamination [17,20,23,24,25,26,29,30,31,32,35,36,37,40,42]. Indeed, cross-contamination may easily arise though the use of contaminated tools and grinding machines. Contamination may occur during the filling of trim boxes or patty forming as these operations usually involve meat from different animals and thus several potential contamination sources may exist [24,30].

Considering storage during post-processing, this step corresponds to the cold storage step at retail. Authors may consider that no temperature abuse occurs during cold storage at slaughterhouse, retail outlet, or during transport to retail [17,25,42]. Predictive microbiology is used to model the possible pathogen growth between the final processing of meat sections and their purchase. This step may also be merged with the distribution chain. Distribution denotes the meat transport-to-home stage. This way, growth models consider temperature abuse associated with cold chain failure. Some authors considered that no microbial growth may occur during transport to retail and subsequent storage [42]. Dayhum et al. [42] studied the outcomes of microbial growth as an alternative scenario. The same assumptions were sometimes made for the cold storage at retail [16] with some authors considering the effects of temperature abuse or freezing on contamination levels [24]. Looking for cross-contamination at retail, Tuominen et al. [40] stated, after solicitation of experts, that this phenomenon is not prone to occur for *Salmonella* spp. as long as the prevalence in beef products remains under 1%.

Considering the distribution step, most studies considered microbial growth that can occur during travel as negligible [23,24,42,44,86]. No contamination or cross-contamination is accounted, but the risk of microbial growth is still considered by several authors since cold chain failure may happen between the distribution of products to the consumer and their cold storage at the consumption place [16,17,24,25,32,33,34,35,36,40,44].

#### 3.3.4. Consumer Practices

The consumer step involves all steps running from the retail store up to meat consumption and includes both meals consumed at home or at collective catering areas. It comprises cold storage, meal preparation, and consumption.

Cold storage at home or catering services is the step where temperature abuse is most likely to occur. This temperature can even be higher during storage at home as it is highly dependent on the current wear condition of the fridge/freezer and considering also that storage places at home are not monitored, especially in the case of domestic fridges. This is the stage where growth modelling is fully and almost systematically taken into account [17,24,32]. In this way, the QMRA led by Kosmider et al. [17] on EHEC and diverse meat products highlighted the distribution (see Section 3.3.3) and storage steps as mainly responsible for microbial growth leading to an illness risk increase.

The preparation step corresponds to the meal preparation through meat handling or mixing with other ingredients, for example, along with meal cooking. This step may involve a high risk of microbial transfer from meat to vegetables and vice versa [19,24,35,38,46] from improperly cleaned hands or from meat to meat if the same cutting media is shared [19]. As an example, Evers et al. [19] considered microbial transfers from meat to vegetables, cutting board, and washed hands. On the other hand, Kosmider [17] stated for its assessment that consumer infection may occur only through consumption of primary contaminated meat instead of secondary contamination. From a general perspective, Signorini and Tarabla [35] compared the impact of industrial and home-prepared burgers on the illness risk associated with EHEC infections and found that storage and preparation conditions at home may lead to almost a tripling of the risk of infection. Considering the cooking stage, predictive microbiology is used here to model the bacterial inactivation due to heating—if no raw meat consumption is assessed, of course. Heating may affect pathogens differently according to the meat configuration considered, as well as the heating method. The vast majority of authors chose to focus on a stove or grill cooking which implies a surface heating of the meat, with the exception of Guillier et al. [41] who distinguished different heating methods. In surface heating conditions, it is possible that the core of the burger or ground meat may not be adequately heated as suggested by some authors [17,21,32,42]. Dayhum et al. [42] estimated that at the coldest point of a patty, only 20% of bacteria (*Salmonella* spp. considered here) are subjected to the heat treatment with an impact of the fat content of the patty on their survival. Tuominen et al. [40] consider that heating at 70 °C totally inactivates *Salmonella* spp. Several authors’ [16,19,34,42] main choice for modelling this inactivation is to consider a heating treatment evenly applied on all the product and so also on all the bacteria population.

It is of importance to consider the fact that modelling all steps occurring between the purchase of meat at retail and consumption at home may be difficult to account for with precision in the exposure assessment as there is no regulation or easy way to carry out sampling, especially at the level of the population of a country [32]. This leads to the use of approximations in these cases, with surveys on part of the population of interest.

### 3.4. Dose/Response Relationships

Some studies performed a hazard characterization to describe the outcomes of a consumer exposure to the pathogen [16,17,20,22,23,24,25,26,29,30,32,33,34,35,36,40,41,42,44,46,48]. A dose-response relationship is expressed as the probability of illness according to the ingested dose. To do so, distribution of health outcomes is observed in groups of humans and animals.

Probability of infection and severity of disease outcomes are different for healthy adults and “sensitive” populations including children under 16, pregnant women, elderly, or immunodeficient people. This statement is especially true when considering *L. monocytogenes* risk assessments for pregnant women [46] or EHEC risks associated with hemolytic uremic syndrome (HUS) for young children [29]. 

For the first models developed, corresponding data could come from controlled human trial studies where volunteers were administered a dose of pathogen to observe the occurrence of illness and its severity [92]. Nowadays, the majority of such studies are done on healthy adult volunteers mainly due to ethical reasons. This fact may tend to lead to an overestimate of the infectious dose compared to the dose that may be applicable in sensitive populations to induce the illness. Moreover, other limits appear. Not all pathogens can be tested, as some are obviously too dangerous in terms of symptoms to be administrated to humans and estimated doses are highly dependent of the strain studied. In order to bypass those hurdles, the vast majority of recent dose-response models tend to start from actual epidemiological data; mainly from well documented outbreaks and try to fit stochastic models accounting for pathogen doses ingested as well as the symptoms found. Stochastic models that are currently the most commonly used are the Beta-Poisson models [16,17,20,21,22,23,24,30,32,33,35,36,42] due to their ability to consider pathogen doses and host susceptibility [93]. Considering *Salmonella* spp., one widely used dose-response model is the one from Teunis et al. [93] based on the Beta-Poisson model fitted on data from 38 *Salmonella* spp. outbreaks with known dose and attack rates and showing high diversity of food sources. In the same vein the model published by Powell et al. [94] for the USDA-FSIS Pathogen Modelling Program [34] is also based on Beta-Poisson equation but fitted on EHEC and *S. dysenteriae* trial data. Contrary to some previous models, these models considered no threshold effect, which means that it considers that the ingestion of at least a single cell can lead to the infection and the illness.

### 3.5. Risk Characterization

Risk characterization corresponds to the final part of QMRA endeavoring to estimate the outcomes of the exposure of consumers to the pathogen and is based on the outputs from the exposure assessment and dose-response relationships parts of the assessment. This step of the QMRA is preferably used by food safety agencies as it gives a picture of the population’s health status. From the papers collected, risk characterization models gave results in the form of incidence, mortality, illness risk, outbreak risk, severity of outcomes (probability of hospitalization), or DALY.

Incidence, as well as mortality or illness and outbreak risks, gave insights on the general state of the population, directly depicting the expected global impact of the contamination. Illness and outbreak risks appeared as straightforward outputs to assess the general health, commonly given per 100,000 persons. Where illness risk illustrated the probability of appearance of at least one new illness case in the general population, outbreak probability [42] described the probability to observe at least two illness cases in this population. Incidence helps to estimate the expected number of new cases brought by the assessed contamination event or possibly avoided by a risk mitigation intervention. This output usually derives from illness risk values multiplied by the population.

On the other side, other metrics used include outcome severity [24,33] and DALYs [16] that are less easy to grasp but carry other useful information, particularly if one also seeks to assess the financial impact of the contamination. Indeed, by knowing the mean cost of a hospitalization for an individual, financial and risk managers might be able to use the probability of hospitalization for a known population to estimate the cost of any contamination during the meat chain. The same way, DALYs can be used to estimate the health impact by integrating in a same output quality and duration of life lost due to illness, expressed in years of life lost in perfect health state.

### 3.6. Modelling Approaches

#### 3.6.1. Top-Down and Bottom-Up Studies

Exposure assessments can be carried out in two ways: starting from the contamination event along the meat chain to model the subsequent exposure for the consumer—known as the bottom-up approach—or from actual epidemiological data to identify the contaminated product—known as the top-down approach. While the latter is often used by national food safety agencies in order to confirm the source of actual reported outbreaks and understand the underlying leading mechanisms, the former is often conducted by scientists during exploratory work to identify the most important contamination risk factors. It is also used by risk managers to assess the impact of Food Safety Objectives (FSO) fixed for the assessed meat chain steps. A FSO corresponds to a threshold frequency or concentration of a microbial hazard to not exceed at the time of consumption (or before the end of product shelf-life) in order to meet a public health goal [95]. These two methods differ slightly from each other in a few aspects, which are illustrated by analyzing two studies on EHEC contaminations.

A bottom-up study conducted by Cummins et al. [31] assessed the prevalence of contamination on beef trimmings and the bacterial count on contaminated trimmings at the end of the slaughter process in Irish abattoirs. To do so, the authors chose to start from EHEC prevalence on hides and feces, with values for hide issued from a parallel study done in order to allow for partial validation of the QMRA and values for feces from the literature. To fill the data gaps not covered by the parallel studies, it was decided—as for almost all bottom-up QMRA studies—to use values from previous studies. Studies used second-order Monte-Carlo simulation with a focus on the dehiding step as the main cross-contamination factor. This simulation method was chosen because it is an effective way to highlight the steps considered. Using this bottom-up approach, Cummins et al. established that the main source of uncertainty when trying to assess the contamination was the sensitivity of the microbial tests used when sampling, followed by the impact of secondary contamination occurring during the dehiding of carcasses, as both prevalence and counts on hides were ranked high. This kind of assessment may provide possible risk reduction measures to risk managers searching for ways to improve food safety objectives.

Considering the top-down approach, the study from Delignette-Muller and Cornu [21,22] investigated an outbreak from 2005, mostly concerning French children under five years of age and associated with the consumption of contaminated frozen ground beef at home. The working group was mandated to carry out a QMRA based on data from the documented 2005 outbreak. This investigation led to the identification of the contaminated meat lot with assessment of the prevalence of contaminated patties as well as enumeration of bacteria on them (all patties being contaminated) along with the incidence of illness; here hemolytic and uremic syndrome (HUS). Due to the fact that the subsequent investigation on the contamination source was carried on up to the retail step with frozen patties, the authors designed their QMRA to focus solely on the retail step and the consumer step within the exposure and risk assessment. Data gaps concerning age-specific consumption frequency, serving size, and cooking preferences were filled by conducting surveys on the French population shortly after the outbreak. Data gaps dealing with microbial destruction by cooking were filled by experiments to obtain missing values. In addition, exposure to the pathogen was assessed with regards to the age of the consumers, as well as the risk for HUS as an outcome. As dose-response models were considered as not suitable to estimate the incidence of HUS cases for children under five, two models were developed in order to reflect the collected epidemiological data. This approach helped to bring the fact that children under five were five times more susceptible to STEC than others. Moreover, this study identified the fact that three of the most used dose-response models, to assess the population between 10 and 16 and designed for general population, tend to underestimate the HUS incidence rates for children under five when considering high contamination levels, like during the outbreak from 2005.

#### 3.6.2. Predictive Microbiology

Predictive microbiology is an essential technique used in QMRA, in particular to carry out exposure assessment while dealing with growth/inactivation phenomena that occur over time [96]. Commonly considered events include cold storage steps at slaughterhouse, at retail, and at the point of consumption, cooking during the preparation step, and cross-contamination events that may occur throughout the farm-to-fork chain. These events can be modelled using different types of predictive microbiology models: microbial growth, inactivation, and transfer models, respectively.

In the case of microbial growth, predictive microbiology models are considered to estimate the increase in the pathogen concentration in the product. These situations mainly depend on temperature during cold storage without freeze (≥4 °C), temperature abuse during storage [16,17,19,20,24,25,26,30,31,32,33,34,35,36,42,44,46,48], or transport times [17,24,33,34]. Models used here can be of a different nature, namely stochastic or deterministic (see Table 2. Deterministic inputs use point estimates whereas stochastic inputs use probability distributions to characterize randomness, variability, and uncertainty [97]. Cummins et al. [31] assumed that EHEC growth at boning hall may be simulated by an increase of 0.33 log_10_. Meanwhile, authors considered that growth occurring during chilling of the carcass could be modelled using the Gompertz equation like other authors have done [17,20,24,25,26,30,32,33,34,35]. Gompertz equations employed here are commonly cited as based on the works from Walls et al. [98] and Marks et al. [99]. Some authors followed other paths with the use of the model from the Pathogen Modelling Program [100] specially developed by the US Department of Agriculture to predict growth and inactivation of pathogens in several environmental conditions. For retail temperature above 0 °C, Smith et al. combined a primary model from Baranyi and Roberts [101] with a secondary model from Tamplin et al. [102], considering as well that bacterial concentration remains stable at temperatures under 0 °C. For his study, Dayhum [42] developed a logistic polynomial regression based on the observation of the growth of *Salmonella* spp. on ground beef and according to 162 combinations of environmental factors, including temperature, pH, or acetic acid.

Incorporating inactivation of pathogens during cooking or freezing can be done by considering both the surface and internal microbial population of the meat piece to be cooked. To do so, various methods have been devised. The easiest one to apply is the logistic [17,19,22,23] and linear [24,32] regression model, but several studies based their inactivation modelling on D and *z* models [19,25,28,34,35,36,46]. One model commonly employed [20,25,28,34,35,36] is the one used for the USDA-FSIS assessment on ground beef from 2001 [34] and developed by Juneja et al. [103]. Developed for combinations of EHEC and ground beef and chicken, this model simulates the one-log_10_ decrease of the bacterial population of interest following an increase of temperature (*z*) for a given cooking time (D). Other authors tried to assess the impact of cooking levels—well done, medium, and rare—on the bacterial population of different meat preparations. For example, Duffy et al. [30] accounted for the impact of the three cooking levels on the contamination levels and prevalence of EHEC in ground beef patties. Inactivation may also occur during cold storage if the temperature is set below 0 °C. To predict this inactivation, Duffy et al. [30] chose to use a distribution representing a concentration decline between 0 log_10_ cfu/g and 3 log_10_ cfu/g, as seen in previously collected data, while the majority of authors assumed no change in concentration.

Cross-contamination along the meat chain can result from contamination of safe meat by contaminated meat and surfaces at several steps along the chain, including at the carcass splitting or meat mincing; through the use of the same machines or cutting boards; and through handling or by mixing different meat lots in boxes during the packaging step. Accounting for these contamination events can be difficult as it depends on a high diversity of parameters from cleaning frequency of the cutting machines, respect of good hygiene practice by handlers, respect of the good hygiene practice plans, and also up to droplet dispersion following rinsing of carcasses.

To take these parameters into account as best as possible and give a realistic assessment of their impact, several methods have been developed. From our collection of papers, it appears that factors from point values, functions, and stochastic simulations have been applied. Evers et al. [19] chose to use a simple parametric function [104] with input data from the literature to consider the cross-contamination that may occur during meat preparation by the consumer. In the same vein, Manyori et al. [38] and Foerster et al. [46] employed factors from several other works considering meat preparation. On the other hand, Tuominen et al. [40] decided to use a simple parametric function developed by Maijala et al. [105] for the poultry meat chain to evaluate secondary contamination occurring during beef slaughter and meat cutting steps. Finally, Signorini et al. [35] and Teagasc [32] chose to estimate the hide-to-carcass cross contamination factor occurring during processing using Beta-distributions based on values from a literature search. 

As stated by Manyori et al. [38] based on the work from Evers and Chardon [106], cross-contamination does not differ with the type of food product assessed as long as preparation methods remain similar. This is helpful in assessing cross-contamination due to the lack of literature concerning cross-contamination of beef carcasses and with pathogens like *Salmonella* spp. Some authors, like Foerster et al. [46] using international data, had to assume that cross-contamination factors do not change between countries as well as between poultry and beef meat preparation for their model. Evers et al. [19] chose to hinder the differences between countries through the use of a factor determined thanks to the study of 30 research articles, still dealing with different meats. Other assumptions made by authors concerning cross-contamination were to assume no or unavoidable pathogen transfers during specific steps. On the other hands, authors may consider hide-to-carcass transfers as inevitable [35] whereas other authors may only consider these transfers—as well as gut-to-carcass transfers as the main routes of cross-contamination [32]. Another assumption that can be made when considering some “worst-case” scenarios is that when an animal is infected, for example by *Salmonella* spp. [40], all meat that could be obtained from the carcass is contaminated. The opposite may apply too if we consider non-infected beef.

#### 3.6.3. Sensitivity and Scenarios Analysis

Once the microbial QMRA model is developed, a sensitivity analysis can be performed to identify variables that are most likely influencing the microbial risk and hence help identify possible interventions to apply. These sensitivity analyses may help risk managers in decision-making by identifying predictive factors of interest.

Sensitivity analyses help to identify steps which will have the greatest impact on the QMRA final outputs, for example prevalence of illness. These analyses can be done in several ways. The first one is the rank-order correlation [20,31,32,34,36,48]. This non-parametric analysis will rank each collected value according to the others in order to rank the correlation between each input value and the studied output through the calculation of their Spearman’s rank order correlation coefficient *r*. This analysis has the advantage of being effective even if inputs and outputs share a complex relationship. Spearman’s rank order correlation led by Cummins et al. [31] showed that sensitivity of the detection test, hide-to-carcass transfer rate, initial hide prevalence, and first decontamination intervention at chilling step had the greatest influence on the final prevalence and counts of EHEC on contaminated trimmings. Another sensitivity analysis technique used in collected articles is the regression analysis [35,37]. This method assumes that there is a relationship between one variable input, all others remaining constant, and the observed output and brings very accurate results if used with additive or subtractive models. By using the Pearson’s correlation coefficient, this method reveals the weight of the studied relationship. Gonzales-Barrón et al. [37] used this method to observe the sensitivity of the *Salmonella* spp. prevalence in beef joints, revealing the contribution of boning, evisceration, and carcass splitting, as well as the relevant efficiency of rinsing if correctly done. In the same way, the analysis highlighted the small influence of the initial pathogen prevalence on hides on the contamination of joints at the end of processing. Brookes et al. [27] chose to use the variance-based Saltelli’s global sensitivity analysis to highlight the most efficient intervention strategies to decrease STEC contaminated prevalence of beef carcasses. This method is able to assess the individual importance of each tested input but also estimates its importance regarding interactions with other inputs.

Models are also used to evaluate the effect of alternative scenarios corresponding to potential mitigation strategies [16,20,24,25,27,28,33,34,43,46,47]. This method mainly consists of evaluating several variations in the initial conditions of the model in order to observe their impact on the final output. The conditions to be varied may range from the bacterial concentration or prevalence of the hide [27,34,35] to the impact of an additional interventions along the meat chain [19,20,24,25,27,28,33,34] to the size of the portion [25]. This method is both useful and the easiest to apply, while considering any relationship already accounted by the QMRA model itself. This analysis is well suited to assess the impact on outputs of changes in inputs that appear to be unrelated to outputs. An example of an application can be the assessment of a microbial reduction achieved through a risk mitigation measure on the pathogen concentration of a product at a given stage of the chain. Brown et al. [28] used this method to assess the impact of intervention strategies on fallen beef carcass at the slaughterhouse in terms of public health, revealing that organic acid rinsing, trimming, or cooking of the carcasses would be more efficient than a water rinsing.

Some authors decided to run correlation analyses in order to describe how changes in the model inputs were linked to changes in the model outputs [33,44]. Finally, Ebel et al. [24] considered sensitivity analysis to highlight variables influencing EHEC contamination of grounded beef meat without naming the method, which we assume to be a correlation analysis, too.

**Figure 5:**
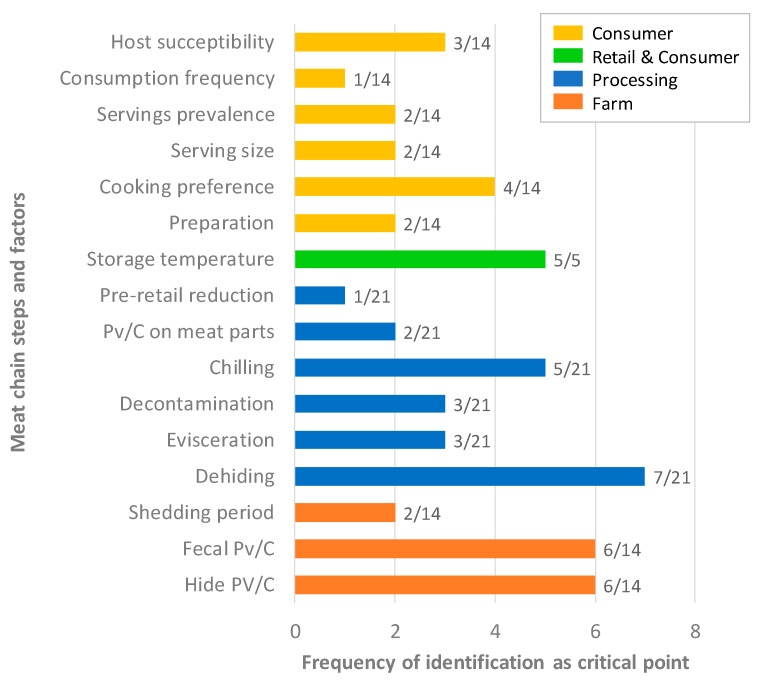
Frequency of identification of steps and factors as critical points for each meat chain stage (Pv/C: prevalence and/or counts). NB: steps or factors were counted only when they were identified within the first five priorities, e.g., dehiding 7/21 means that this step was considered in the sensitivity analysis of 21 QMRAs and identified seven times in the first five priorities.

Sensitivity analyses highlight several critical steps, revealing their contribution to the final contamination of the beef products and their public health impact, and the interventions that may be conducted to hinder them (Figure 5). The three most critical action levers to reduce overall microbial health risks that emerge from the analysis of the articles are the dehiding steps and associated handling measures [24,27,31,32,34,36,37], followed by prevalence and counts on hide [16,20,27,34,36,43] and in feces [24,30,31,32,35,43]. Thus, primary contamination at the farm level before its arrival at the slaughterhouse is crucial to be controlled [18,31]. Other factors identified were related to temperature: the chilling steps during processing [16,24,35,48] and the storage temperature at retail, home, and during transit from retail to home [30,33,34,35,36]. These factors would mainly be related to problems of temperature abuse and shelf life that could occur between the end of processing and meat consumption. These factors are succeeded by cooking preferences and the associated inactivation [30,34,36,48] and then by a set of factors consisting of the evisceration of animals [27,36,37] and the subsequent decontamination step [24,34,37], as well as the consumer’s susceptibility to the pathogen [20,36,46]. While the importance of cooking preferences is strongly associated with its influence on the inactivation of pathogens on the surface and in the center of the meat, the smaller impact of the evisceration stage may be related to the fact that it is one of the most recognized stages for contamination and, therefore, one of the most regulated and controlled in the chain.

The combination of mitigation strategies was also suggested to get a more efficient way to reduce the risk as illustrated by Brown et al. [28]. They suggested the combination of trimming and cooking treatments as more efficient than any of them applied individually to try to avoid additional illnesses associated with the contamination of fallen beef carcasses. Several factors identified through sensitivity analysis may be hard to achieve, like reducing the prevalence of infection at the farm [27] or prophylaxis by giving more information to consumers concerning the importance of properly cooking meat which may appear to be already sufficiently done. Furthermore, Smith et al. [36] concluded that combining pre-harvest and processing interventions may greatly help to achieve a significant risk reduction, while a report from USDA-FSIS [34] concluded a better risk mitigation from the combination of microbiostasis during storage and achieving a five log_10_ microbial concentration reduction during cooking to allow for a risk reduction of four log_10_ during the meal preparation.

#### 3.6.4. Validation of Models

Due to the use of models, the given outputs are based on a limited set of input factors which only constitute the essential part of the studied situation. To ensure robustness of the model results, authors may seek to validate them, as done for 11 of the collected studies.

Results validation can be done following different methods. In several cases, results of studies were validated with data from surveys done at specific meat chain steps [16,17,21,22,31,32,34,36,37,39]. A part of these data are issued from past studies for a couple of papers, with Cummins et al. [31] using data from a study from 2006 and Tuominen et al. [39] validating its assessment of *Salmonella* spp. risk associated with imported beef products on national and international prevalence as assessed in 1999. Other studies tend to carry out samplings in parallel with their work. As an example, Nauta et al. [16] used data from another study done in parallel to establish the STEC prevalence in raw steak and the incidence of associated illnesses in Dutch population [107]. The study by Teagasc [32] had main outputs from two modules of the QMRA—prevalence and concentration on beef trimmings as well as beef products at retail—which were validated against data derived from microbial samplings along the corresponding meat chain steps. Finally, outputs of the dose-response relationship can be validated with data from actual outbreaks. This was done for outputs of the work done by Delignette-Muller and Cornu for AFSSA [21,22] that were compared to the corresponding outputs from six EHEC outbreaks associated with diverse products across the world.

Some studies that did not rely on data from the field have chosen to use statistical validation [38,41]. In this case, model outputs are validated using other dose-response models to compare the results in terms of incidence, prevalence, or DALYs associated with the corresponding illnesses. Thus, the salmonellosis incidence estimated by Guillier et al. [41] was compared to the corresponding uncertainty distributions obtained from the exposure calculated during the QMRA. Concerning the work of Manyori et al. on the impact of *Salmonella* spp. contamination of beef products on Zambian population health [38], intermediate and final outputs of the deterministic Swift QMRA model used here were compared to results from a full-scale QMRA accounting for *Campylobacter* spp. contamination of chicken products as “a measure of relative risk.”

#### 3.6.5. Uncertainties and Variability

Modelling the behavior of pathogens in meat along the whole farm-to-fork chain requires a large amount of data of varying nature: prevalence of pathogens in feces or on hide or cross-contamination risk during mincing, for example. Generating these data requires extensive monitoring and expertise at each step of the farm-to-fork chain. The first limitation is the fact that a considerable amount of data gaps are present and hinder the possibility of making models that are as close as possible to reality [27]. These data gaps are linked to information that was not or could not be retrieved or modelled. Brookes et al. [27], when assessing microbial risks associated with EHEC and Australian chilled carcasses, stated that the most important information gap they struggled with was data on the impact of the distribution of EHEC fecal and hide concentrations on the probabilities of transfer to carcasses with the resulting concentration distribution on their surface. Concerning transport of meat from the slaughterhouse to the retail outlet, Dayhum [42] had to consider no microbial growth due to a lack of data on applied temperatures and possible abuse. Those issues were associated with two indisputable aspects of model inputs: variability and uncertainty. Hence, the impact of the distribution of hidden contamination on the hide-to-carcass transfer of pathogens is an element of variability as it would heavily vary according to animals and could eventually result in an underestimation of the contamination. Ignoring microbial growth during transport brings a source of uncertainty, as considering the temperature abuse as non-existent is an assumption that can alter the relevance of the results to reality. Variability can be better described but cannot be reduced with an additional amount of data as variability is inherent to any biological phenomenon, whereas the uncertainty can be reduced by obtaining more data or knowledge.

Moreover, in the presence of a large amount of data, models have to consider the random nature of several phenomena, i.e., variability occurring along the meat chain. To overcome these hurdles, models commonly follow two modelling methodologies in order to assess the risk: the deterministic and the stochastic methodologies (see Table 2). The deterministic approach is based on the use of point estimate as inputs for the different inputs of the model. This approach has the advantage of giving a simple response under the form of a value, for example a mean prevalence, without having to use statistical distributions. Among collected works, this approach was used by the authors of five research articles [19,38,44,46]. Evers et al. [19] studied, for instance, the impact of pre-retail processing, home storage, and preparation of raw meat from different animals on the exposure of the Dutch population to EHEC using bacterial prevalence and concentration point values on and in meat as inputs. In the same way, Jeong et al. [44] assessed the illness risk associated with consumption of raw beef from retail in South Korea using mean prevalence and initial contamination of the meat parts, with those data being from a sampling campaign. One major drawback of this method is the fact that it can only give restricted insights through its outputs.

A stochastic approach swaps point value inputs for probability distributions and uses Monte-Carlo simulation. These distributions consider variability and uncertainty of the results but require more expertise to implement. Accounting for these aspects of the results helps to better consider randomness of the process. From our research, a total of 31 papers used stochastic methods with a total of 32 models used [46] which represents 86.1% of the collected works reflecting the appeal to specialists for this method. Assessing the illness risk and the mortality per serving and years associated with the consumption of US ground beef and due to EHEC infections, USDA-FSIS [34] based its model on a Bayesian distribution of the fecal bacterial prevalence at the farm. On the other hand, Foerster et al. [46] modelled the probability of listeriosis following Chilean chicken and beef consumption using three QMRA models; one deterministic and two other stochastic ones. Whereas the deterministic model used initial *Listeria monocytogenes* concentration in meat as well as the contamination prevalence as the main input, the stochastic ones used the variability and/or uncertainty ranges of these parameters as inputs. The majority of papers using stochastic modelling have also used Monte-Carlo simulation to assess the risk exposure with precision. By randomly sampling a high number of values, this method helps to make objective assessments accounting for the inherent randomness of the input. Moreover, this method can account for the highly exposed populations that represent a fraction of the general population. To simulate the values to be sampled, one can use Bayesian distributions of them as well as Poisson, Beta, Weibull-Gamma, and binomial ones [16,17,20,21,22,23,24,25,26,29,31,32,33,35,39,40,41,48].

## 4. Discussion

Considering variability and uncertainty is essential in QMRA and was implemented in different ways in the selected beef models, the use of stochastic processes with Monte-Carlo simulation was the most common option taken compared with the use of deterministic inputs. It enabled to capture variability and uncertainty of inputs implemented by using probability distributions and to perform sensitivity analysis to identify the most influential variables in a model, to provide a better understanding and interpretation of the analysis, and to identify data gaps and then prioritize future research [108,109,110,111]. Several data gaps and sources of unquantified uncertainties were identified. For instance, Brookes et al. [27] identified factors such as the impact of the distribution of contamination on hides and in feces on the transfer probability onto the carcass as one important data gap to fill. Likewise, Cummins et al. [31] and Ebel et al. [24] highlighted the sensitivity of the diagnostic tests done on animals and the influence of the season on the prevalence of illness at the farm. Several authors also raised questions concerning the possible spreading of contamination between carcasses when washing them with water [32,36,37].

To reduce public health risks associated with beef meat consumption, specific risk mitigation strategies must be put in place. If dealing with EHEC or *Salmonella* spp., for example, the main relevant action levers place themselves between the farm and the chilling of the carcass, which encompasses the majority of the entry points for the contamination as well as numerous data gaps (dispersion of pathogens on hide, shedding condition of the animal, pathogen dispersion during dehiding, etc.). If dealing with *L. monocytogenes*, efforts have to be put on storage conditions at retail and at home, with information to the consumer instead of focusing on the slaughterhouse.

As pointed out by our analysis, it appears that the majority of collected studies focused on modelling only a selection of steps along the whole farm-to-fork chain. The decision to focus on certain steps is justified by the initial risk assessment question which can be very focused, or by the fact that some microorganisms can be controlled by targeting specific steps. For instance, main mitigation strategies to control *Listeria monocytogenes* focus on storage steps, while strategies for EHEC focus on the reduction of its prevalence before slaughter and the reduction of cross-contamination on the product. Similarly, Nauta et al. [16] established that measures such as product control for STEC and microbial growth at retail may show little interest as microbial contamination predicted by their model is rather low at this step and growth appears unlikely to occur. Additionally, authors stated that further information about better cooking to consumers would also be of low interest. Another example is the potential cross-contamination during transportation from the farm to the slaughterhouse or during holding in the lairage [27]. They are difficult to model but can provide useful insights to assess the impact of good cleaning procedures for transport trucks and at the lairage, as well as an emphasis on the impact of the length of the transit and lairage time on the cattle’s stress and the subsequent changes on its pathogen shedding. Consequently, it is not necessary to model the whole farm-to-fork chain when focusing on specific questions.

Finally, the present critical analysis has highlighted a large heterogeneity of models used in each study, so on a broader scale it is not possible to rank risk mitigation strategies in terms of effectiveness or hazards in terms of priority [112] when they have not been evaluated in the same model. Nevertheless, we can identify the different risk mitigation strategies highlighted during the sensitivity analyses of QMRA studies; they were identified in Figure 5. The steps related to the slaughterhouse were the most investigated strategies to reduce contamination with the dehiding, and chilling of carcasses identified as a priority. Then, the management of on-farm transfers of faecal contamination remains a top priority in order to limit the introduction of pathogens into the processing chain. Finally, the storage temperature during transportation or at home, as well as the cooking methods used, play an important role.

Another challenge is to answer a new public health question applying previous QMRA. Using a modular process risk model approach could facilitate the reuse of all or parts of models as it divides the food pathway in a sequence of modules, each describing the change of level and/or prevalence of microorganism due to growth, inactivation, partitioning, mixing, removal, and cross-contamination [113]. Indeed, QMRA is a promising method to support the decision-making process in food safety [114] and its use could be facilitated by the harmonization of the QMRA approach [13] which would avoid duplication of efforts [115]. 

## 5. Conclusions

This study aimed to synthetize and analyze available QMRA studies performed for beef meat consumption using a systematic approach to collect exhaustive QMRA studies. They were synthesized and methods used were analyzed. This work will provide to scientists and risk managers an overview of current beef QMRAs studies available with an insight on research gaps identified.

Our analysis highlighted that QMRA is a well-established approach and an essential technique to estimate the potential impact of decisions measures on public health outcomes. Moreover, these tools can help to identify most of steps at risk in the meat chain before an actual contamination event that may lead to major outcomes and therefore avoid it. To do so, assessments may need a lot of data according to the way they are conducted, but it remains possible to focus the work on several steps following the matrix-pathogen couple assessed and reduce the cost in terms of data needed. Although QMRA can be done using data from the literature, it becomes even more relevant when based on inputs from the field, including actual surveys on disease prevalence in animals or pathogen concentration in samples. Modelling methods vary with the use of both deterministic and stochastic models, with a wide variety of methods for the latter and a greater interest from the scientific community.

On a broader view, collected studies tend to emphasize the main contribution of contamination at the farm level, with contaminated feces or hide, to the chilling of the carcass, and the contaminations associated with carcass dehiding, evisceration, or splitting. This statement can encourage risk managers to focus on these steps on the farm and at the slaughterhouse. However, conditions of preparation that can induce bacterial growth or contaminations can still occur in the subsequent steps as cross-contamination may happen during preparation of the meal, for example. In fact, consumer behavior appears to be an important driver. Sensitivity analyses done by authors on the microbial levels and prevalence of servings brought to light the important impact of the cooking method [20,32,33,34,36,48], the storage temperature at home [32,33,34,35,36,46], and even the consumption frequency of beef meat [44]. Nevertheless, reducing the contamination levels at the farm remains one of the most impacting factors [20,24,31,32,34,35,36,113].

As outputs of these assessments are based on mathematical models, some authors decided to add a validation step to their study to assess uncertainty or variability of the outputs. This has been done through comparison of intermediate outputs to field data, sampling or documented outbreaks, or application of statistical methods like uncertainty distributions. This process can appear as essential for some QMRA users to bring more robustness and “reality” to the outputs.

In addition to the predictive aspect, sensitivity analyses can add value to the QMRA as it may highlight some meat chain steps overlooked by models or maybe considered as insufficiently monitored. Pre-emptive identification of these steps is one of the most valuable advantages of these analyses, as well as one of the easiest-to-grasp outputs for risk managers. This method can indeed generate the final output of risk-ranking tools and provide the list of action levers amongst the steps of the beef meat chain that have the most impact on final product contamination at time of consumption.

Nevertheless, models can only provide estimations with a level of accuracy that depends on quality of data used as the input and also on their consistency. Several data gaps have been identified throughout the meat chain that could highlight more pertinent outputs from the models, but data extraction from the corresponding steps can be difficult or even almost impossible in several cases. Proposed risk mitigation interventions for steps identified as “at risk” can also be unrealistic and hence the hazard can remain. Eventually, QMRA outputs can reveal themselves as hard to manipulate and to translate as interventions, which renders this assessment less useful than expected.

In conclusion, QMRA is a very powerful tool providing valuable insights to underpin risk-management decisions [116] in a risk-based approach [117,118]. In order to improve its efficiency and facilitate its use and reuse, it is necessary to develop a tool to harmonize the various models available in the literature and enable risk assessments on different matrix-pathogen combinations.

## Figures and Tables

**Table 1:** Summary of beef quantitative microbial risk assessment (QMRA) models collected.

Pathogen	Productt	Country	Population	Objective	Ref.
*Escherichia coli EHEC* (*n* = 21)	Beef meat	The Netherlands	All	Exposure assessment, health burden, interventions	[16]
		UK	All	Exposure assessment for beef, sheep and pig meats	[17]
	Beef preparations	The Netherlands	All	Exposure assessment from meat of different animals	[19]
	Burger	Canada/North America	All	Process assessment	[20]
		France	<16 years	Evaluation of illness risk following outbreak	[21,22]
			All	Impact of cooking preferences on illness risk	[23]
		North America	All	Food-chain assessment; health burden assessment	[24]
			All	Evaluation of illness risk from Australian beef	[25]
		Scotland	All	Risk assessment of transmission pathways to humans	[26]
	Carcasses	Australia	All	Assessment of interventions	[27]
		North America	All	Assessment of interventions for fallen carcasses	[28]
		Scotland	All	Exposure assessment of carcass processing	[18]
	Ground beef	France	<16 years	Process assessment	[29]
		Ireland	All	Food chain assessment	[30,31]
			All	Illness risk from consumption Risk management	[32]
		North America	<5 years, >5 years	Illness risk from consumption	[33]
			All	Illness risk from consumption	[34]
		Argentina	<5 years, adult	Illness risk from consumption	[35]
		Canada	All	Intervention ranking Assessment of diverse meat types	[36]
*Salmonella* spp. (*n* = 7)	Beef meat	Brazil	All	Meta-analysis-based exposure assessment	[37]
		Zambia	All	Assessment of increasing beef consumption on public health	[38]
	Beef products	Finland	All	Assessment of imported beef and additional guarantees	[39]
			All	Assessment of impact of performance objectives and microbiological criteria	[40]
	Burgers	France	All	Outbreak investigation	[41]
	Ground beef	France	All	Evaluation of illness risk	[42]
		North America	All	Contribution of deep tissue lymph nodes to meat contamination and interventions	[43]
*Campylobacter* spp. (*n* = 1)	Raw beef	South Korea	All	Risk assessment of raw beef offal	[44]
*Cryptosporidium parvum* (*n* = 1)	Beef	North America	-	Estimation of beef-attributed daily shedding	[45]
*Listeria monocytogenes* (*n* = 1)	Beef meat	Chile	Susceptible	Estimation of illness probability from beef and chicken consumption	[46]
Bovine Spongiform Encephalopathy (*n* = 1)	Cattle	UK	All	Assessment of the impact of risk-reduction measures	[47]
*Taenia saginata* (*n* = 1)	Beef	Australia	All	Adaptation of model to national context, impact of interventions	[48]

**Table 2:** Main inputs, outputs and methods observed among the collected QMRA models (Pv: prevalence; C: count and/or concentration; P_ill_: probability of illness; P_out_: probability of outbreak; In: incidence; DALY: disability-adjusted life years expectancy; Mo: mortality; *S*: stochastic model; *D*: deterministic model).

Pathogen	Ref	Inputs	Outputs	Model	Validation	Predictive Microbiology	Dose-Response	Sensitivity Analysis
*Escherichia coli* (EHEC)	[16]	Pv	Pv; C P_ill_; In; DALY	*S*	Literature	Growth Inactivation	Beta-Poisson	Dependency
	[17]	Pv	Pv; C P_ill_	*S*	Literature	Growth Inactivation	Beta-binomial	-
	[18]	Pv	Pv	*S*	-	-	-	-
	[19]	Pv; C	C Ex	*D*	-	Growth Inactivation	-	Dependency
	[20]	Pv; C	Ex; Dose P_ill_; Mo	*S*	-	Growth Inactivation	Beta-binomial; Beta-Poisson	Rank order correlation
	[21,22]	C	Pv; C P_ill_; In	*S*	Literature	Inactivation	[0–5] and [5–10] Exponential; exponential-Poisson [10–16] Beta-Poisson	-
	[23]	Pv; C	In	*S*	-	Inactivation	Beta-Poisson	-
	[24]	Pv	Pv; C P_ill_	*S*	-	Growth Inactivation	Beta-Poisson	Correlation
	[25]	Pv	Pv P_ill_; In	*S*	-	-	Beta-binomial	Dependency
	[26]	Pv; C	Ex; Dose P_ill_; In	*S*	-	-	Beta-Binomial	-
	[27]	Pv	Pv	*S*	-	-	-	Saltelli’s method
	[28]	Probability of carcass falling; C; In	In	*S*	-	Inactivation	-	Dependency
	[29]	Pv; C	p_ill_	*S*	-	Inactivation	Exponential	
	[30,31]	Pv	Pv; C	*S*	Sampling	Growth	Beta-Poisson	Rank order correlation
	[32]	Pv; C	Pv; C P_ill_	*S*	Samplings	Growth Inactivation	Envelope model	Rank order correlation
	[33]	Pv	Pv P_ill_	*S*		Growth	Beta-Poisson	Correlation Dependency
	[34]	Pv	Pv; C P_ill_; In; Mo	*S*	Literature	Growth Inactivation	Beta-Poisson	Rank order correlation Dependency
	[35]	Pv; C	Pv; C P_ill_	*S*		Growth Inactivation	Beta-Poisson	Regression
	[36]	C	Dose P_ill_	*S*	Sampling	Growth Inactivation	Beta-binomial	Rank order correlation
*Salmonella* spp.	[37]	Pv	Pv	*S*	Survey	-	-	Regression
	[38]	Pv; C	Ex; C In	*D*	Literature	-	-	-
	[39]	Pv	Pv	*S*	Literature	-	-	-
	[40]	Pv	Pv P_ill_; In	*S*	-	Inactivation	Beta-Poisson	-
	[41]	C	Dose In	*S*	Literature	Inactivation	Beta-Poisson	-
	[42]	Pv; C	Ex; Dose P_ill_; P_out_	*S*	-	Growth Inactivation	Beta-Poisson	-
	[43]	Pv; C	Pv; C	*S*	-	-	-	Dependency
*Campylobacter* spp.	[44]	Pv; C	Pv/; C P_ill_	*D*		Growth	Beta-Poisson	Correlation analysis
*Cryptosporidium parvum*	[45]	Pv; In	Total shedding	*S*				
*L. monocytogenes*	[46]	C	In	*D; S*		Growth Inactivation	Exponential, Weibull-Gamma	Dependency
Bovine Spongiform Encephalopathy	[47]	Pv; In Infectivity	Ex P_ill_	*S*	-	-	-	Dependency
*Taenia saginata*	[48]	Pv National production	Dose P_ill_	*S*		Inactivation	Beta distribution	Rank-order correlation
